# When Does Directional Reflectance Matter? Evaluating BRDF Effects in Plant Canopy Light Simulations

**DOI:** 10.3390/plants15071043

**Published:** 2026-03-27

**Authors:** Jens Balasus, Felix Wirth, Alexander Herzog, Tran Quoc Khanh

**Affiliations:** Laboratory of Adaptive Lighting Systems and Visual Processing, Technical University of Darmstadt, Hochschulstr. 4a, 64289 Darmstadt, Germany

**Keywords:** ray-tracing simulation, optical sensor simulation, spectral BRDF modeling, cucumber, light simulation

## Abstract

Virtual plant models combined with ray-tracing simulations are an established tool for evaluating plant–light interactions. Current approaches often simplify leaf surface properties by assuming diffuse reflectance behavior, despite experimental evidence that leaf reflectance is direction-dependent across much of the visible spectrum. This study investigates whether incorporating measured, spectrally resolved and direction-dependent (BRDF) reflectance properties into these models affects simulation outcomes. Using virtual 3D cucumber (*Cucumis sativus*) plant models with PhongShader-based optical leaf characteristics for BRDF consideration, light absorption and local photon flux densities were simulated under a wide range of lighting conditions, including diffuse and directed sunlight scenarios. While total light absorption at the leaf level is only marginally affected (mean absolute percentage error, MAPE < 2%), spectral distortions in leaf surroundings, especially under direct light, exceeded 8% in the blue wavelength range. Beyond their relevance for estimating photosynthetic rates, such distortions directly affect the spectral composition within the canopy, which is particularly critical in greenhouse applications where optical sensors are used to monitor spectral ratios and, therefore, require the accurate prior simulation of canopy light conditions. This is particularly relevant for setups with directional artificial lighting. The findings suggest that BRDF modeling is not critical for calculating photosynthetic rates under most conditions, but is required in spectral analyses or for optimizing artificial lighting designs.

## 1. Introduction

Light distribution within plant canopies is a key determinant of photon absorption and thus photosynthesis, morphogenesis and ultimately crop yield. Over the last decade, functional–structural plant models (FSPMs) coupled with Monte-Carlo ray tracing have become a central tool to investigate plant–light interactions in silico, because they link realistic 3D plant architectures and illumination to simulated light interception and, consequently, to plant-level photosynthetic activity under diverse environmental lighting conditions. In parallel, experimental work in controlled environments has emphasized the importance of measured spectral ratios such as red:far-red or blue:red within greenhouses for characterizing lighting strategies (e.g., [1,2,3]), which underscores their role for both applied greenhouse management and the calibration of modeling approaches.

Many canopy-scale studies compute light interception, carbon gain, or light-use efficiency from spectrally resolved leaf reflectance and transmittance (ρ(λ), τ(λ)) but assume Lambertian (diffuse) scattering at the leaf surface (e.g., tomato and cucumber canopies under sun and greenhouse lighting [4,5,6,7]). In contrast to this simplification, it has long been established that leaf reflectance varies with wavelength and illumination–view geometry. By the 1970s, Breece and Holmes had measured bidirectional reflectance of soybean and maize leaves to report directional reflectance behavior [8]. Subsequent work described the direction-dependent reflection behavior by the bidirectional reflectance distribution function (BRDF, [9]), which specifies how reflected radiance depends on illumination and viewing directions. Further studies reported the leaf-surface dependent specular components across species ([10], pp. 101–105) and indicate that typical greenhouse crops such as cucumber or tomato have been under-represented. More recently, physically motivated models for tree leaves were developed [11,12]. In other works of the authors, gaps for cucumber leaf optics were addressed, and spectral-dependent BRDF properties were determined [13]. Despite this progress, spectral BRDF data have rarely been integrated into whole-plant, spectrally resolved ray-tracing with realistic geometry and environmental variability.

In summary, the key limitations of prior work include: (1) most canopy simulations assume Lambertian leaf surfaces without evaluating the resulting error, (2) existing BRDF studies have been limited to leaf-level characterization without propagating directional effects to whole-plant simulations and (3) systematic evaluations across a range of lighting conditions, particularly those relevant to controlled-environment agriculture, are lacking.

Physically based microfacet models, such as those described by Roth et al. [12] and Bousquet et al. [14], provide a detailed physical description of surface scattering and can, in principle, represent the measured BRDF of plant leaves with high accuracy. However, these models are not implemented in commonly used virtual plant simulation frameworks, which limits their applicability for the type of large-scale, spectrally resolved simulations conducted in this study. Cieslak et al. [15] demonstrated the feasibility of incorporating BRDF into ray tracing, but not in spectrally resolved, full-plant simulations. It, therefore, remains unclear under which lighting conditions the spectral and angular nature of leaf reflectance affects simulation outcomes, particularly in scenarios with strong direct components that are common in controlled-environment agriculture.

While Chelle [16] found that diffuse modeling errors can be negligible in dense maize canopies, that analysis did not spectrally resolve BRDF and focused on dense maize under natural light. In this study, light simulations were conducted using a distribution function adapted to the BRDF of maize leaves. The results indicated minor differences between simulations employing the measured BRDF and those assuming diffuse reflectance, with the magnitude of these differences depending on solar elevation. Based on these findings, Chelle concluded that, for very dense crop canopies, a diffuse reflectance model may be sufficient. However, it should be noted that maize exhibits architectural traits that differ substantially from those of common greenhouse crops. Therefore, the transferability of these conclusions to other species or canopy types remains uncertain.

Two classes of models are commonly used to study plant–light interactions: (1) within-canopy FSPM ray-tracing frameworks [17] and (2) scene- and sensor-oriented 3D radiative-transfer models [18,19,20]. While both allow the implementation of leaf-level reflectance characteristics, their primary outputs differ. The remote-sensing applications are commonly focused on image synthesis and top-of-canopy radiance retrieval, rather than within-canopy light analysis. In contrast, virtual plant applications range from quantifying the impact of global radiation on photosynthetic rates [6,21,22,23,24,25,26] and analyzing how plant architectural traits modulate light interception [4] to optimizing artificial lighting layouts in controlled-environment agriculture [5,27,28,29]. Representative studies are summarized in Table 1. While several approaches rely on spectrally resolved reflectance and transmittance (e.g., [30,31,32]), no study incorporates spectrally resolved BRDF effectsand their influence on (1) organ-level absorption and (2) the local spectral photon field surrounding leaves under directional light. That is the gap we address in this work.

In this study, we employed the spectrally resolved BRDF of cucumber leaves, parameterized in our previous work using the empirical Phong reflection model [13]. This choice was motivated by its native implementation in the GroIMP simulation framework and its compatibility with per-wavelength fitting based on measured reflectance data. The use of the Phong model enables an explicit consideration of direction-dependent reflectance effects in large-scale, spectrally resolved plant-canopy simulations to investigate the effect of the diffuse approximation on simulation outcomes.

To this end, we parameterized the measured spectral BRDF of cucumber leaves in GroIMP [40] using the implemented PhongShader and evaluated virtual plants across diffuse and directional lighting scenarios. Consistent with prior reports, the measured cucumber BRDF exhibits more pronounced directionality in the blue (450 nm) and red (660 nm) ranges and is closer to diffuse in the green (550 nm) and far-red (730 nm) ranges [11,12,41].

This study contributes:

- A quantitative comparison of spectral BRDF modeling versus a diffuse approximation with respect to light absorption and local photosynthetic photon flux density (PPFD).
- A systematic evaluation across diffuse and directional lighting scenarios.
- Criteria that delineate when BRDF modeling is beneficial and when it is negligible in canopy-scale simulations.

## 2. Materials and Methods

### 2.1. Plant Material and Data Structure

Young cucumber plants (*Cucumis sativus*, Saladin F1 hybrids, 28–30 days old, five-leaf stage) were selected for their manageable size and relevance in greenhouse horticulture as experimental plants. At this growth stage, leaf overlap occurs, enabling the analysis of inter-leaf shading and directional reflectance effects. Twelve plants (mean height 0.212 ± 0.03 m) were selected for 3D modeling, with the capture of typical leaf arrangements, curvature and internode structure. Optical measurements were performed on a separate set of plants. The leaf order increased from the lowermost fully expanded leaf (order one) to the youngest (order five).

Since a primary application of virtual plant models is the simulation of horticultural crops under natural and artificial lighting conditions, we adopted a spectral band division that corresponds to ratios commonly used to characterize horticultural luminaires [42]. This scheme has also been employed in virtual plant simulations by Hitz [43]. Accordingly, the blue range was defined as 440 nm to 500 nm, the green as 500 nm to 600 nm, the red as 600 nm to 700 nm and the far-red as 700 nm to 740 nm. For brevity, wavelength ranges are omitted hereafter. Kahlen et al. [33] further emphasized the distinct role of wavelengths around 700 nm due to their specific photomorphogenetic relevance. While simulations were performed at 30 spectral bins, the results were aggregated into the four bands for analysis. For applications such as remote sensing, in which narrow spectral bands are of particular importance, different aggregation strategies may be required.

**Figure 1:**
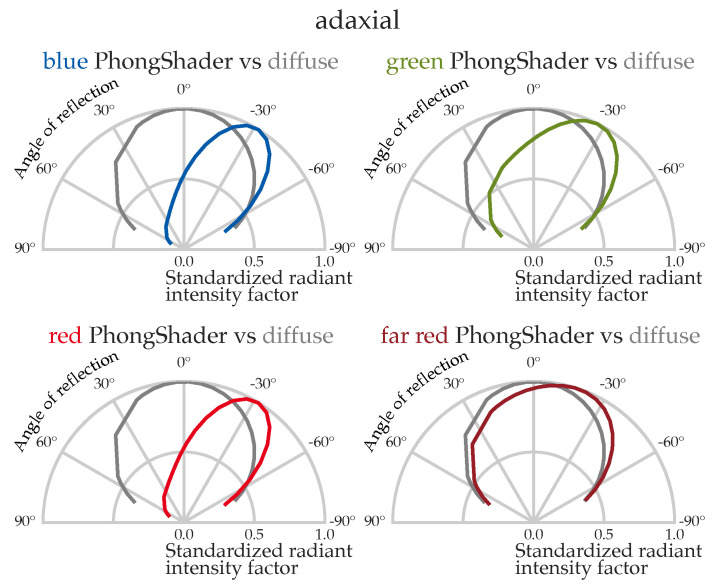
Spectral PhongShader radiant intensity distribution for an exemplary cucumber leaf surface, compared to a diffuse reflectance model. All curves are normalized to their respective maximum reflectance values. The angle of light incidence is fixed at 40°. Radiant intensity was derived from BRDF measurements and weighted by the cosine of the reflection angle to account for the projected solid angle in hemispherical space (following $I=cos\left(\theta_{r}\right)\;\cdot \;f\left(\theta_{r},\theta_{i}\right)$ with radiant intensity *I*, angle of reflection $\theta_{r}$, and angle of incident radiation $\theta_{i}$ and BRDF *f*); this leads to a shift in the specular peak towards the normal, with the magnitude of the shift depending on the specular-to-diffuse ratio, which varies spectrally. For more details on this, see [13] or [9]. Spectral values represent the blue (450 nm), green (555 nm), red (660 nm) and far-red (730 nm) reflectance characteristics of the adaxial leaf surface.

The spectral BRDFs of both the adaxial and abaxial sides of all leaves from a representative cucumber plant were measured using a gonioreflectometer setup, as illustrated in Figure 1 and described in detail by [13]. PhongShader parameters were fitted per wavelength using non-linear least squares optimization (LMFIT package in Python (version 3.9.0) [44]). They were adjusted to the hemispherical spectral reflectance values while validating energy conservation for each wavelength. This yielded a spectral set of direction-dependent shader parameters for GroIMP. The spectral NRMSE between the fitted Phong model and the measured reflectance distributions is detailed in Figure A2. Across the analyzed wavelength bands, the Phong model achieves a minimum mean NRMSE of 0.104 in the far-red (700–740 nm) and a maximum of 0.500 in the blue spectral range (<500 nm). In comparison, the diffuse model exhibits significantly higher deviations, with NRMSE values ranging from 0.195 (far-red) to 0.951 (blue). Comprehensive data for all spectral ranges and model orders are summarized in Table A3. Furthermore, the deviation in energy conservation, which constitutes the upper bound for the systematic error within the simulation, is discussed in Section 2.3.

### 2.2. Creation of Geometry Models

Geometric plant models were created using photogrammetry-based 3D scanning [45] workflow. Each plant was imaged from 360° using a rotating turntable and four 16 megapixel-RGB-cameras (IMX519, Arducam, Hong Kong). COLMAP software [46,47] was used to calculate point clouds from the image sets, followed by manual cleanup and skeletonization in Meshlab [48]. Leaf surfaces were reconstructed using characteristic leaf points, as described by [49]. Additionally, the positions of the nodes and internodes were extracted. Using these datapoints, virtual plants were reconstructed in GroIMP using cylinders and triangles with PolygonMesh.

### 2.3. Simulation Setup

Global lighting was represented by a hemispherical distribution of 108 directed light sources (9 altitude × 12 azimuthal divisions), following the solar light model used by [5].

The integrated lighting scenarios vary the ratio of diffuse and direct irradiance in 10% increments while keeping the total global irradiance constant ($E_{\mathrm{global}}=E_{\mathrm{diffuse}}+E_{\mathrm{direct}}=\mathrm{const}$). The azimuths of the directed light source were set to 0°, 90°, 180° and 270°, and the altitudes to 10° to 90° in 10° steps, resulting in 360 unique combinations. These configurations enable generalized conclusions across a wide range of realistic lighting conditions, ranging from natural lighting conditions, such as the mixture of diffuse and direct light at various positions, to artificial lighting conditions with purely directional light as found in horticultural luminaires.

**Figure 2:**
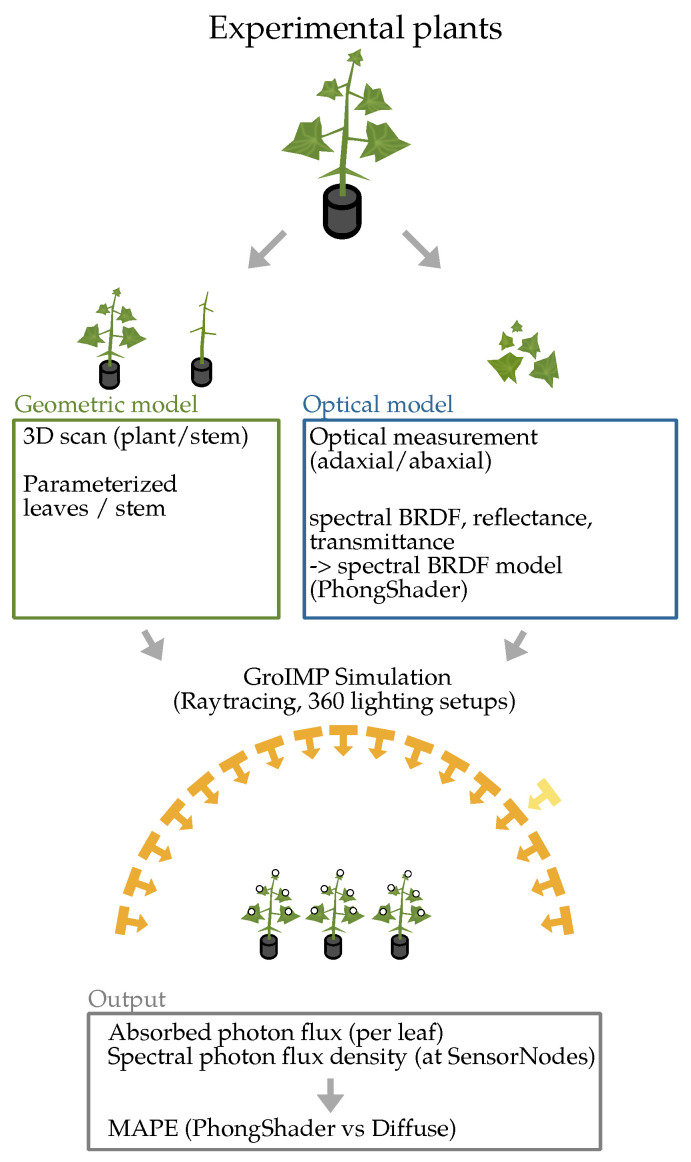
Overview of the experimental pipeline with all subprocesses.

The D65 standard illuminant spectrum (CIE standard representing average daylight) was applied uniformly across all simulations. The spectral photon flux absorbed by the leaf areas was measured. Additionally, SensorNodes (virtual detector elements that record local spectral irradiance in the simulation environment) were placed 5 mm above and below the leaf surfaces to measure local photon flux density. An overview of the experimental pipeline is given in Figure 2.

**Figure A1:**
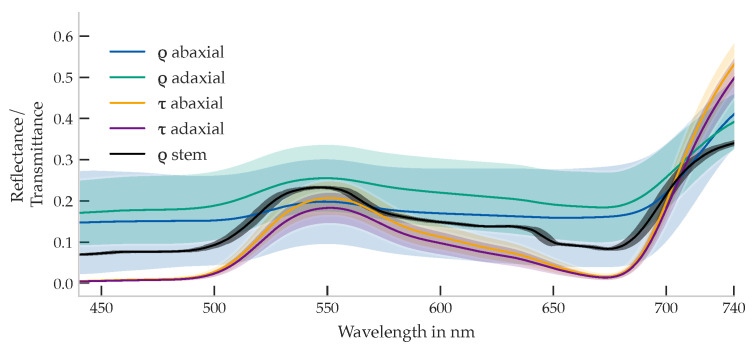
Averaged spectral reflectance and transmittance of both leaf sides with enveloping standard deviation. Calculated from all plant data sets. (*n* = 34). Additionally shown is the spectral reflectance of the stem, calculated from measurements of 18 stem parts of three different plants.

**Figure A2:**
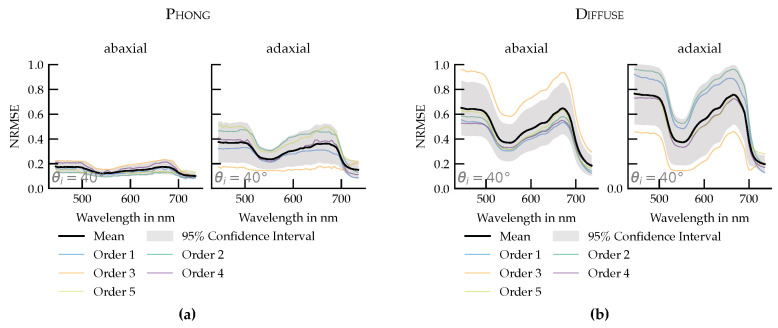
(**a**) Normalized root mean square error (NRMSE) between Phong model-simulated univariate reflectance distribution functions and measured spectral univariate reflectance distribution functions for the abaxial and adaxial leaf surfaces. (**b**) NRMSE between the measured spectral univariate reflectance distribution functions and a purely diffuse reflection model, scaled to match the peak intensity for the abaxial and adaxial leaf surfaces. In both subfigures, the parameters are fitted at 40°. Figure adapted from Balasus et al. [13].

The evaluation is based on the hypothesis that the impact of spectral BRDF modeling on simulation outcomes depends on the angle of light incidence and the proportion of direct radiation. Due to the simulation and the associated abstraction, the following assumptions and limitations apply to the experiments. These are partly due to measurement techniques and partly deliberately chosen to ensure minimal influence outside the properties under investigation:

- Model basis: Virtual plant models are reconstructed from measured geometric and optical data. Leaf geometry follows [49]. Uniform stem thicknesses and reflectances (Figure A1, Table A1, Appendix A) are applied, stems are non-transmitting.
- Environment: Plants are in unbounded space with a zero-reflectance ground to eliminate back-reflection. Minor 3D scan inaccuracies do not affect relative internal comparisons. The D65 spectrum is used globally.
- Optical properties: Leaf transmission is assumed to diffuse [14]. BRDFs are spatially uniform per leaf side (no intra-leaf variation).
- Comparison approach: To isolate BRDF effects, each simulation compares results using spectral PhongShaders versus spectrally equivalent diffuse shaders.
- Energy conservation: Fitted Phong shader deviations (sum of reflectance, transmittance, absorbance) are 0.8% at 40° incidence. At grazing angles (>70°), deviations increase to 2.91–5.68%, requiring individual interpretation.

**Figure 3:**
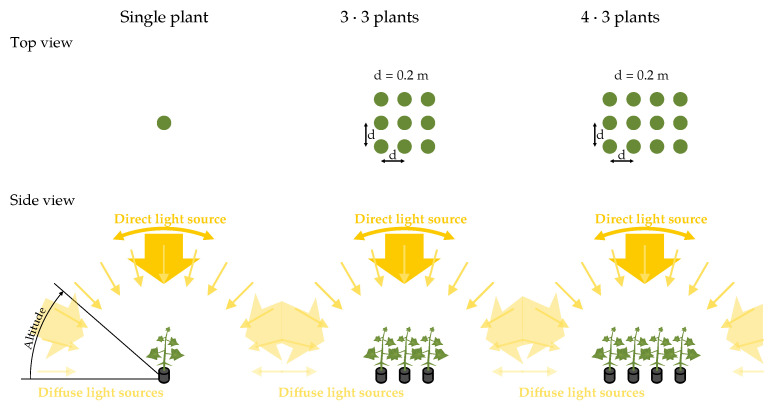
Sketch of the experimental setup and light variations. The hemisphere is divided into nine segments at the zenith and twelve segments azimuthally, totaling 108 segments, each with a directed light source at its center. A directed light source, variable in azimuth and zenith angle, represents direct sunlight.

**Figure 4:**
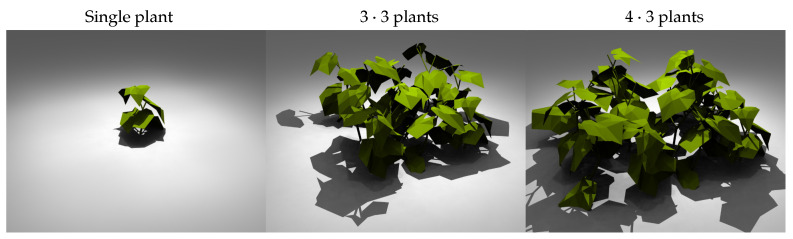
Renderings of plant arrangements in GroIMP. The leaves are uniformly colored for better visualization and do not correspond to the spectral PhongShaders. Unlike in the simulation, a ground is included. Shadows are emphasized.

Three canopy configurations were simulated (Figure 3): (1) single plants, (2) a three-by-three square grid and (3) a four by three grid (Figure 3). The leaf area index (LAI) with the spacing of 0.2 m is 4.625±1. In dense grid arrangements the amount of multiple reflected light received by the central plants is supposed to be higher, increasing the influence of BRDF. Renderings of the scenes are given in Figure 4. The four-by-three arrangement was specifically chosen to introduce asymmetry into the canopy layout: while the symmetric three-by-three grid provides identical surroundings for all central plants, the additional row in the four by three configuration exposes the central plants to a non-symmetric neighborhood, allowing us to test whether BRDF effects are sensitive to asymmetric inter-plant reflections.

The verification of existing multiple reflections is done by deactivating ray reflection in the simulation settings, so only light directly emitted from the light sources is measured (see [30]).

Simulations were performed with $2\;\times \;10^{9}$ rays (the maximum supported by GroIMP) per run, distributed over 30 spectral bins from 440 nm to 740 nm. This ray count exceeds the 20 million recommended by [50]. Reflection depth is set to 50 to limit computation time (based on [43] and verified in pre-experiments for the given scenarios).

Two quantities are determined in each simulation:

1. Absorbed spectral photon flux by unit leaf area
2. Spectral photon flux density at three reference points on the adaxial and abaxial leaf sides. The spectral photon flux density is determined from the spectral irradiance.

All simulations are run three times with a different random set of rays. Subsequently, the simulations for each scenario are averaged to minimize stochastic variation.

The simulation evaluation in this work focuses on three aspects: (1) the proportion of indirect light, (2) the total absorbed photon flux per leaf (light interception) and (3) the spectral photon flux density in the surrounding leaf environment.

## 3. Results

### 3.1. Indirect Light Proportion

To evaluate the potential influence of BRDF effects, the proportion of indirect light was first quantified under varying conditions. Indirect light was calculated by comparing two simulations: one with and one without reflection modeling. Disabling reflections isolates the direct component, allowing the indirect share to be derived by calculating its contribution:

(1) $$\eta =\left(1-\frac{E_{p,w/o\mathrm{multi}\mathrm{reflection}}}{E_{p,w\mathrm{multi}\mathrm{reflection}}}\right)$$

where $E_{p,w/o\mathrm{multi}\mathrm{reflection}}$ is the absorbed photon flux density without reflections, and $E_{p,w\mathrm{multi}\mathrm{reflection}}$ is the one with activated reflections. The calculated indirect light contributions are differentiated by scenarios and by the centrally positioned plants in the three-by-three and four-by-three arrangements.

**Figure 5:**
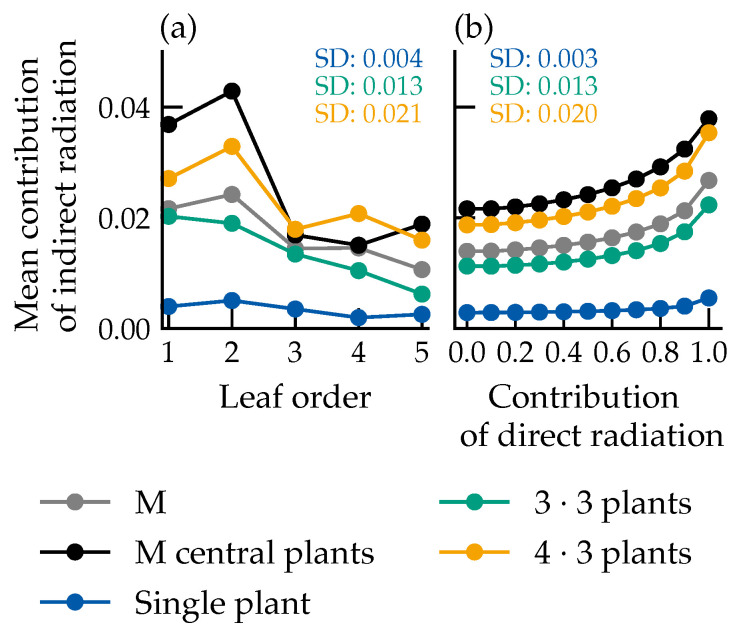
Average indirect contribution of absorbed photon flux for the different experimental groups. The contribution is determined using Equation (1). The values of the central plants are determined based on three plants. The average standard deviation is given for the individual scenarios. M: arithmetic mean across all plants in the respective arrangement; M central plants: mean of centrally positioned plants in grid arrangements; SD: standard deviation. Number of samples used for each data point: (**a**) leaf order: M (*n* = 19,008), M central plants (*n* = 2376), single plant (*n* = 2376), 3·3 plants (*n* = 7128), 4·3 plants (*n* = 9504). (**b**) Contribution of direct radiation: M (*n* = 8640), M central plants (*n* = 1080), single plant (*n* = 1080), 3·3 plants (*n* = 3240), 4·3 plants (*n* = 4320).

For centrally positioned plants, the indirect contribution peaked at approximately 4% in lower leaf orders and decreased to about 2% toward the canopy top, partly due to reflections from the fourth leaf onto the abaxial side of the fifth (Figure 5a). This trend held across all layouts, with negligible indirect light in single-plant scenarios. The indirect contribution increased with the proportion of direct radiation (Figure 5b).

**Figure 6:**
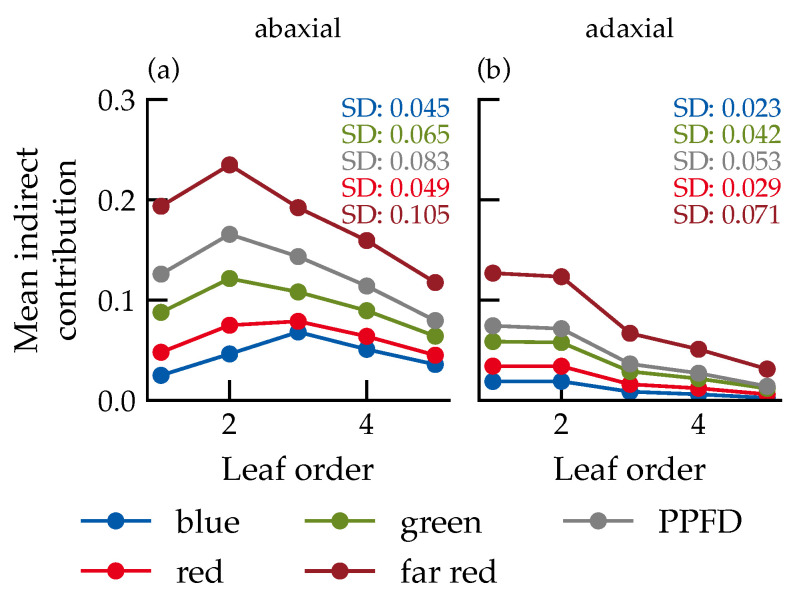
Average indirect contribution of the different spectral ranges, depending on leaf order for the abaxial (**a**) and adaxial (**b**) leaf sides. The data is based on the spectral photon flux densities at the virtual sensors in the surrounding of the leaves of the respective leaf order. Data represent the mean across all canopy configurations. Number of samples per data point: *n* = 28,458.

Near-leaf sensors revealed higher indirect proportions on the abaxial side (Figure 6), with spectral contributions ranked as descending far-red, green, red, blue, which is consistent with the spectral reflectance and transmittance properties of leaves.

### 3.2. Influence of Spectral and Directional Surface Properties on Absorbed Photon Flux

In light simulations, the absorbed photon flux is often used to estimate ${\mathrm{CO}}_{2}$ assimilation at the leaf level. Since no widely accepted spectrally resolved photosynthesis model is available, absorption is integrated over the full spectrum. To verify the statistical significance of the differences between the datasets, a paired *t*-test was conducted for the overall data (t = 117.8, df = 95, *p* < 0.0001).

The analysis is based on the mean absolute percentage error (MAPE, Equation (2)), which is determined for the respective subgroups:

(2) $$\mathrm{MAPE}=\frac{1}{n}\sum_{1}^{n}\frac{\left|\phi_{p,\mathrm{phong}}-\phi_{p,\mathrm{diffuse}}\right|}{\phi_{p,\mathrm{diffuse}}}$$

where $\phi_{p,\mathrm{phong}}$ denotes the absorbed photon flux density per leaf, simulated using the PhongShader and $\phi_{p,\mathrm{diffuse}}$ refers to the result using the diffuse reflectance model. This ensures that negative and positive deviations do not cancel each other out. The same equation is applied to the spectral photon flux density measured at the SensorNodes.

**Figure 7:**
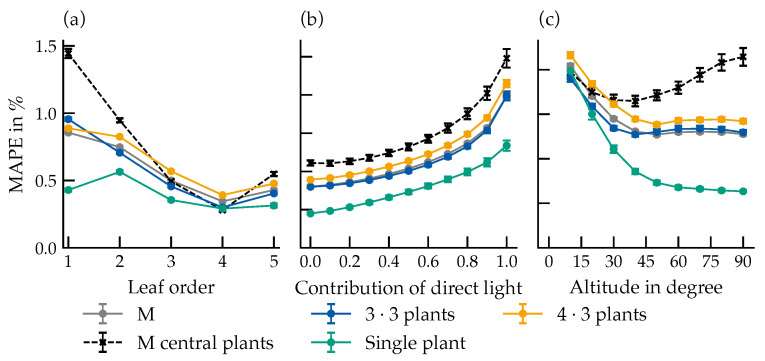
Presentation of MAPE in % over leaf order (**a**), proportion of direct radiation (**b**) and altitude (**c**). M: arithmetic mean across all plants in the respective arrangement; M central plants: mean of centrally positioned plants in grid arrangements. Number of samples used for each data point: (**a**) *n* = 19,008, (**b**) *n* = 8640, (**c**) = 10,560. Error bars represent the standard error (SE). Where not visible, they are smaller than the data markers.

For the integrated absorbed photon flux, maximum MAPE values reached approximately 1.5% in the lower-order leaves of the three-by-three setting (Figure 7a), decreasing steadily with an increasing leaf order. Across all arrangements, single plants showed the lowest MAPE (0.5%), while central plants in denser configurations exhibited higher deviations. MAPE values increased with the proportion of direct radiation (Figure 7b).

### 3.3. Influence on the Spectral Photon Flux Density in Leaf Surroundings

Beyond leaf absorption, the spectral composition of the light environment near the leaf surfaces is critical, especially in the context of optical sensor simulations. To assess this, the spectral photon flux density detected by the adaxially and abaxially placed SensorNodes was evaluated. Paired *t*-tests confirmed statistically significant differences between the datasets across all evaluated spectral bands (blue, green, red, far-red) and the total PPFD (*t*-values ranging from 261.8 to 412.5, df = 285, *p* < 0.0001).

#### 3.3.1. Spectral Composition Depending on Leaf Order

The mean relative contribution of each spectral band is defined as the ratio of the photon flux density in that band to the total photon flux density across all four bands, averaged over all evaluated scenarios.

**Figure 8:**
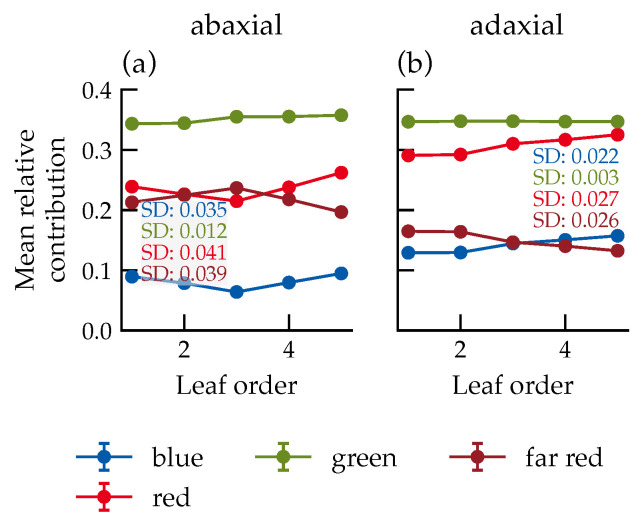
Contributions of several spectral ranges to the total photon flux density over leaf order for the adaxial (**a**) and abaxial (**b**) leaf sides. For clarity regarding order dependence, only values with a proportion of direct radiation from the light source greater than 50% are shown. Values are determined using the PhongShader. Data represent the mean across all canopy configurations. Number of samples per data point: *n* = 28,458. Error bars represent the standard error (SE). Where not visible, they are smaller than the data markers.

For direct light contributions above 50%, green light dominated the total photon flux (34%), followed by red and far-red, while blue contributed 5% to 15%, depending on the leaf order (Figure 8). Spectral composition was largely stable across leaf orders, with only minor shifts on the abaxial side.

#### 3.3.2. Influence on Spectral Ranges

The calculation is performed using Equation (2), where the respective photon flux densities for diffuse reflection and the spectral PhongShader are used instead of the photon flux.

**Figure 9:**
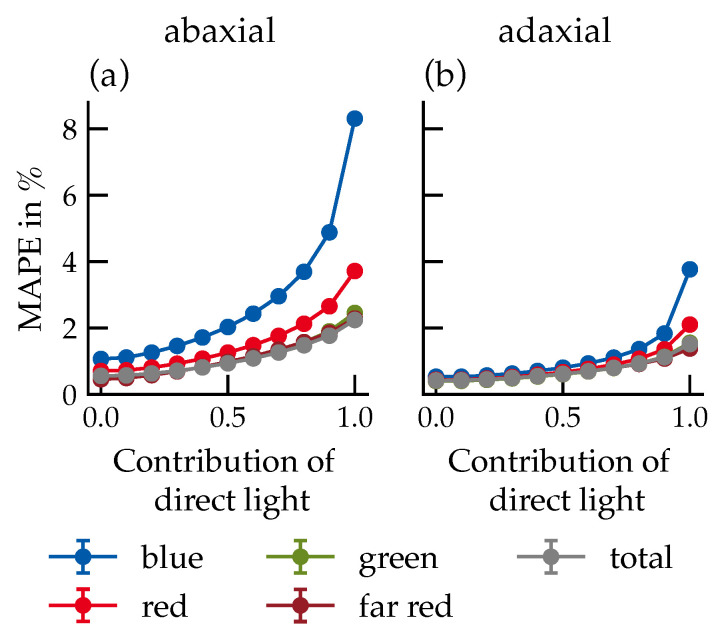
MAPE of different spectral ranges, plotted against the proportion of direct light from the light source for the abaxial (**a**) and adaxial (**b**) leaf sides. Data represent the mean across all canopy configurations. Number of samples per data point: *n* = 12,960. Error bars represent the standard error (SE). Where not visible, they are smaller than the data markers.

The MAPE increased with the proportion of direct light for all spectral ranges, most prominently for blue light on the abaxial side, reaching 8.3% under exclusively direct illumination (Figure 9).

**Figure 10:**
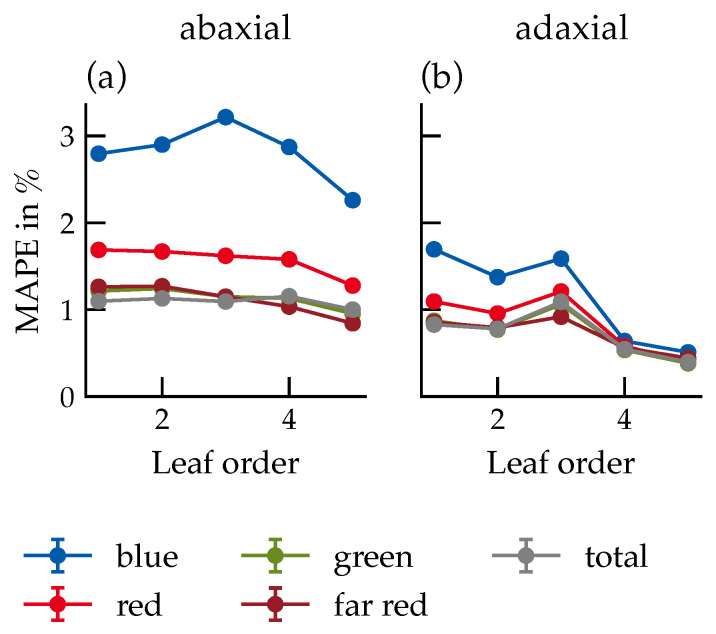
MAPE of photon flux densities in different spectral ranges depending on leaf order for the abaxial (**a**) and adaxial (**b**) leaf sides. The data is based on the measured photon flux densities of the virtual sensors in the surrounding of the leaves of the respective leaf order. Data represent the mean across all canopy configurations. Number of samples per data point: *n* = 28,512. Error bars represent the standard error (SE). Where not visible, they are smaller than the data markers.

Leaf order had a weaker influence on MAPE than direct light fraction (Figure 10), with the blue range again showing the largest deviations (3.1%). A localized anomaly in third-order leaves may reflect variations in specular components or sensor geometry.

Table 2 summarizes the MAPE distribution by spectral range, leaf side and direct radiation proportion. The 90%-quantile confirms that blue-range deviations are the most severe: 17.81% (abaxial) and 11.33% (adaxial). Red-range errors were approximately half as large, while green, far-red and total values remained below 5%.

**Figure 11:**
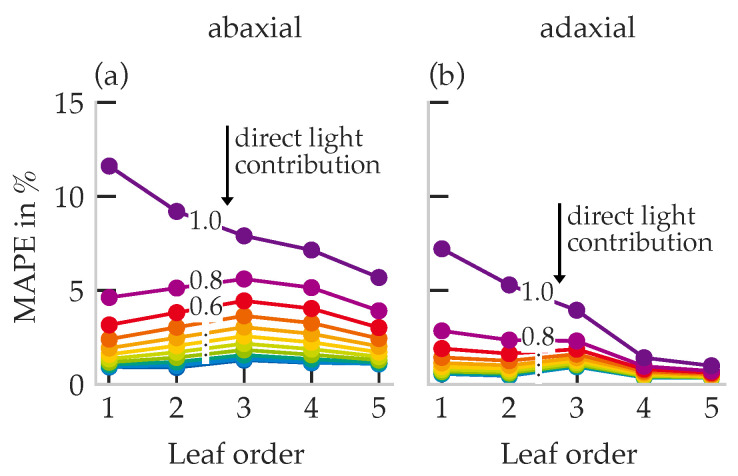
MAPE for all data points in the blue spectral range, plotted over leaf order for the abaxial (**a**) and adaxial (**b**) leaf sides. The plots show the trends for different proportions of direct radiation, varying between 0% and 100% in 10% increments. Number of samples per data point: *n* = 2592.

In the blue range under 100% direct light, abaxial MAPE reached 10% for first-order leaves and decreased with canopy depth; adaxial errors were on average 7% lower (Figure 11).

### 3.4. Summary of Maximum MAPE

The proportion of indirect light was generally low (≤4%) and strongly dependent on canopy density and light direction. Spectral photon flux composition was dominated by green, red, and far-red wavelengths, while blue contributed only 5% to 15%, thereby limiting its effect on total absorption. Table 3 summarizes the maximum mean absolute percentage errors (MAPE) for both absorbed photon flux and the local photon flux density ($E_{p}$) across spectral ranges, leaf sides, and orders.

Across all canopy configurations, absorption-level deviations due to BRDF modeling remained consistently below 1.5%, irrespective of direct light contribution or leaf order. By contrast, much higher deviations were observed for the blue spectral range in the local leaf surroundings under conditions of high directional light, with MAPE values exceeding 8% abaxially and 3% adaxially. Deviations for green, red, and far-red remained considerably lower, all below 4%. This underscores that, for most natural and mixed lighting scenarios, a diffuse model is sufficient for estimating leaf-level assimilation. However, for applications involving high proportions of directional blue light, such as LED-based systems, explicit BRDF modeling may be required.

## 4. Discussion

### 4.1. Behavior of Indirect Light Proportion

The spectral ordering of indirect light contributions (far-red > green > red > blue) is consistent with the wavelength-dependent reflectance and transmittance of cucumber leaves. Because most leaves remain directly exposed in these young canopies, multiple-reflection contributions are limited, supporting the physical plausibility of the simulation. A limitation of this study is that only five-leaf-stage cucumber plants were simulated; more mature canopies with stronger mutual shading may exhibit different BRDF effects due to a higher amount of indirect radiation.

### 4.2. Effect on Absorbed Photon Flux

In the absorbed photon flux simulation, the highest values were measured in the lower-order leaves of the three-by-three setting and were decreasing steadily with an increasing leaf order. This trend mirrors the distribution of indirect light previously observed.

Across all groups, central plants in the denser configuration indicated the highest deviation of the MAPE, indicating that BRDF-related absorption errors are amplified by canopy shading and inter-leaf reflection.

The altitude of the directed light source had no discernible effect on indirect radiation but clearly influenced the leaf absorption, especially for the non-central plants.

Since photosynthesis scales approximately linearly with photon flux density below saturation, the observed deviations could directly affect the calculated assimilation rates. However, even under directional lighting, the maximum MAPE (1.5%) is particularly low, suggesting that BRDF modeling is not critical for assimilation estimates within small plant populations. Notably, this error lies within the deviation range of the Phong reflectance model, which is 0.8% at an incidence angle of 40° but can be as high as 5.68% for steep angles at 85° (see Section 2.3).

These findings suggest that, under natural sunlight (including diffuse and direct components), BRDF effects on leaf-level light absorption are minor. However, in denser plantings or under artificial lighting with strong directionality, these effects could be amplified. Additionally, the low transmittance of blue and red light and their high specularity within leaves reduces the BRDF’s influence in those spectral regions, further minimizing its effect on total absorption.

### 4.3. Effect on Leaf Surroundings

Because blue light constitutes only 5% to 15% of the total photon flux under a D65 spectrum, even substantial relative errors in this range have a limited impact on total photon flux density.

BRDF effects scale non-linearly with direct light fraction (Figure 9): deviations between 50% and 100% direct radiation substantially exceed those between 0% and 50%. This reflects the stronger specular components in the blue and red ranges compared to the more diffuse green and far-red ranges. These spectral differences in directionality are consistent with wavelength-dependent absorption by leaf pigments (primarily chlorophyll), which reduces the diffuse scattering component in the blue and red ranges while leaving the green and far-red ranges, where pigment absorption is low, dominated by diffuse subsurface scattering [10,11].

Overall, BRDF effects influence spectral photon flux density more than absorption, particularly for blue light under directional illumination (MAPE > 17%; Table 2), while the total photon flux remains stable due to green, red and far-red dominance.

These findings are particularly relevant for simulations involving spectrally sensitive sensors or directional artificial lighting, for which accurate spectral rendering is required. In such contexts, simplified diffuse models may underestimate or misrepresent the local light distribution, potentially leading to systematic errors in virtual sensor readings or lighting control algorithms. Incorporating BRDF modeling into these simulations could, therefore, improve predictive accuracy, especially in controlled environments using narrow-angle and spectrally tailored light sources. Furthermore, the use of the empirical Phong model, while implemented in the GroIMP rendering engine, represents a simplification of the physical scattering process. Physically based microfacet models (e.g., Cook-Torrance [51]) could provide more accurate angular extrapolation, particularly at incidence angles far from the 40° calibration angle.

## 5. Conclusions

This study evaluated the impact of incorporating a spectral BRDF model (via Phong shading) compared to a diffuse approximation in plant light simulations, using virtual cucumber canopies under various lighting conditions. The results demonstrate that the necessity of BRDF modeling depends on the intended application of the simulation.

In practical terms, this means that, for the majority of daylight and mixed-light scenarios in plant modeling and greenhouse applications, the added complexity of explicit BRDF modeling does not substantially improve assimilation or total absorption values. Regarding light absorption for photosynthesis estimation, the findings suggest that a diffuse reflectance model is sufficient for large-scale canopy simulations. The observed mean absolute percentage error (MAPE) for total absorbed photon flux remained consistently below 1.5%, even in dense canopy arrangements. Since the error introduced by the diffuse simplification is within the range of uncertainty of the fitting accuracy of the Phong model itself, the added computational complexity of BRDF modeling provides negligible benefits for estimating plant-level photon absorption.

In scenarios involving higher blue-light content, such as certain LED spectra, the relative impact of BRDF modeling on spectral photon flux may increase and should be reevaluated. BRDF-related deviations in the local spectral environment were substantially larger than in absorption. Under full directional light, blue light errors exceeded 17.8% on the abaxial leaf side, with implications for the simulation of optical sensors and spectral control systems in artificial lighting setups.

An examination of the error distribution shows that, particularly with exclusively direct light, large errors can occur. For the blue spectral range, the MAPE is greater than 17.81% (abaxial) or 11.33% (adaxial) for 10% of the data points. These deviations cannot be explained by the errors of the Phong model.

Future modeling workflows should consider spectral BRDF effects, particularly in simulations using collimated light, such as from LEDs or high direct sun contributions, where directional errors are highest. Additionally, the experimental validation of the simulated spectral distributions using spectroradiometer measurements within real canopies represents an important next step. While it is not possible to switch between different reflectance characteristics in real-world experiments, the predicted photon flux distributions under field conditions should be verified.

For field-scale canopy simulations, where edge effects cannot be mitigated by restricting the analysis to central plants, periodic boundary conditions should be considered to quantify BRDF-related errors without confounding finite-arrangement artifacts.

## Figures and Tables

**Table 1:** Overview of light simulations with virtual plants that determine: LI—light interception; LUE—light use efficiency; PS—Photosynthesis; and $E_{p}$—photon flux density. Abbreviations: HPS—high-pressure sodium lamp; r—red; b—blue; g—green; fr—far red; ρ—reflectance; τ—transmittance; and α—absorptance. The subscript for photosynthetically active radiation (PAR) refers to values integrated over the PAR spectral range; if the subscript is missing, the spectral range was not specified.

Source	Plant Type	Light Sources	Optical Properties	Simulated/Calculated Variable
[33]	Cucumber	Sun	$\rho_{\mathrm{PAR}}$,$\tau_{\mathrm{PAR}}$, $\rho_{\mathrm{FR}}$,$\tau_{\mathrm{FR}}$	Leaf Phototropism
[6]	Cucumber	Sun	$\rho_{\mathrm{PAR}}$,$\tau_{\mathrm{PAR}}$	LUE, PS
[21]	Cucumber	Sun	$\rho_{\mathrm{PAR}}$,$\tau_{\mathrm{PAR}}$	LI, PS
[22]	Cucumber	Sun	$\rho_{\mathrm{PAR}}$,$\tau_{\mathrm{PAR}}$	LI
[27]	Cut Rose	Sun, HPS	ρ,τ, 3 ranges	PS rate, LI
[34]	Grapevine	Sun	$\rho_{\mathrm{PAR}}$,$\tau_{\mathrm{PAR}}$	LI
[28]	Kale	UV-B LED	-	Radiation Interception
[35]	Kiwi	Sun	α	-
[29]	Lettuce	b,r LED	ρ(λ),τ(λ)	LUE, PS
[36]	Mango	Sun, Plasma	ρ(λ),τ(λ)	LI, PS
[23]	Pepper	Sun	ρ,τ	LI, PS
[24]	Pepper	Sun	ρ(λ),τ(λ)	LI, PS
[25]	Pepper	Sun	ρ,τ	LI, Leaf and Plant PS, LUE
[30]	Soybean	Sun	ρ(λ),τ(λ)	$E_{p}$
[31]	Soybean	LED	ρ(λ),τ(λ)	$E_{p}\left(\lambda \right)$
[26]	Tomato	Sun	ρ,τ	LI, PS
[4]	Tomato	Sun	ρ,τ	LI, Leaf and Plant PS
[5]	Tomato	Sun, HPS, LED	$\rho_{\mathrm{PAR}}$,$\tau_{\mathrm{PAR}}$	Light distribution, PS
[32]	Tomato	b,g,r LED	ρ(λ),τ(λ)	LUE, PS
[37]	Tomato	HPS	ρ(λ),τ(λ)	LI, PS
[38]	Tomato	Sun, Greenhouse Coating	$\rho_{\mathrm{PAR}}$,$\tau_{\mathrm{PAR}}$	LI
[39]	Tomato	Red LED	$\rho_{\mathrm{PAR}}$,$\tau_{\mathrm{PAR}}$	$E_{p}$, absorbed $E_{p}$
[7]	Tomato	Sun, Greenhouse Coating	ρ(λ),τ(λ)	LUE, Light distribution, absorbed $E_{p}$

**Table 2:** Distribution of the MAPE (in %) between the simulation with considered BRDF using a Phong shader and diffuse reflection. Data are presented as mean (M) ± standard error (SE), alongside the 25%, 50%, and 90% quantiles (Q25, Q50, Q90). The sample size is *n* = 12,960 per evaluated condition (column).

		MAPE in Percent					
Dir. Part		Abaxial			Adaxial		
		0.0	0.5	1.0	0.0	0.5	1.0
total	M	0.56 ± <0.01	0.94 ± 0.01	2.24 ± 0.02	0.42 ± <0.01	0.61 ± 0.01	1.52 ± 0.02
	Q25	0.23	0.30	0.60	0.17	0.21	0.32
	Q50	0.51	0.70	1.53	0.33	0.44	0.76
	Q90	1.01	2.02	5.03	0.82	1.31	3.83
blue	M	1.07 ± 0.01	2.03 ± 0.02	8.31 ± 0.09	0.53 ± 0.01	0.80 ± 0.01	3.77 ± 0.07
	Q25	0.36	0.47	1.66	0.17	0.22	0.39
	Q50	0.79	1.31	5.73	0.38	0.49	1.01
	Q90	2.30	4.69	17.81	1.00	1.77	11.33
green	M	0.52 ± <0.01	0.96 ± 0.01	2.45 ± 0.02	0.40 ± <0.01	0.61 ± 0.01	1.55 ± 0.02
	Q25	0.22	0.31	0.62	0.16	0.20	0.31
	Q50	0.45	0.71	1.62	0.32	0.42	0.74
	Q90	0.95	2.09	5.64	0.81	1.30	4.03
red	M	0.70 ± 0.01	1.26 ± 0.01	3.71 ± 0.04	0.44 ± <0.01	0.67 ± 0.01	2.10 ± 0.03
	Q25	0.27	0.37	0.85	0.15	0.20	0.35
	Q50	0.58	0.89	2.39	0.33	0.45	0.84
	Q90	1.49	2.78	8.46	0.89	1.44	5.75
far-red	M	0.45 ± <0.01	0.98 ± 0.01	2.29 ± 0.02	0.42 ± <0.01	0.62 ± 0.01	1.38 ± 0.02
	Q25	0.19	0.33	0.61	0.20	0.22	0.33
	Q50	0.40	0.74	1.53	0.37	0.46	0.73
	Q90	0.87	2.12	5.21	0.78	1.33	3.46

**Table 3:** Maximum MAPE for absorbed photon flux and $E_{p}$ depending on leaf side, direct radiation contribution and leaf order.

Leaf Side		Direct Radiation Contribution			Leaf Order	
		Abaxial		Adaxial	Abaxial	Adaxial
Absorbed photon flux			1.24%		1.44%	
$E_{p}$ in leaf surrounding	Blue	8.31%		3.77%	3.22%	1.7%
	Green	2.45%		1.55%	1.24%	1.06%
	Red	3.72%		2.10%	1.69%	1.21%
	Far-red	2.29%		1.38%	1.27%	0.92%
	Total	2.24%		1.52%	1.15%	1.09%

## Data Availability

The original contributions presented in this study are included in the article. Further inquiries can be directed to the corresponding author.

## Appendix A

### Appendix A.1

**Table A1:** Mean diameter of the stems and petioles in mm *n* = 3.

	Diameter in mm				
Leaf order	**1**	**2**	**3**	**4**	**5**
Stem	8.95	8.78	6.66	5.74	5.12
Petiole	4.79	5.74	5.53	5.08	4.78

**Table A2:** Mean leaf areas and their standard deviation of the leaves of the modeled plants. The leaf areas were determined based on the parameterized plants in GroIMP. The mean height of the plants was 0.212 m with an SD of 0.03 m *n* = 12.

	Leaf Rank				
	**1**	**2**	**3**	**4**	**5**
Mean leaf area in m^2^	0.037	0.053	0.042	0.034	0.019
Standard deviation (area)	0.005	0.010	0.012	0.006	0.007
Mean inclination angle in degrees	37.596	54.530	43.777	43.128	38.043
Standard deviation (inclination)	13.375	16.893	16.510	20.620	16.734

**Table A3:** Mean NRMSE at 40° for Phong and diffuse reflection models at the used wavelength bands.

Model	Order	Wavelength Band (nm)			
		<500	500–600	600–700	700–740
Phong					
abaxial	Mean	0.174	0.137	0.159	0.110
	Order 1	0.161	0.124	0.144	0.091
	Order 2	0.129	0.104	0.120	0.091
	Order 3	0.223	0.176	0.213	0.125
	Order 4	0.208	0.151	0.185	0.104
	Order 5	0.148	0.128	0.131	0.138
adaxial	Mean	0.370	0.280	0.337	0.176
	Order 1	0.324	0.260	0.296	0.122
	Order 2	0.464	0.369	0.440	0.191
	Order 3	0.171	0.147	0.162	0.182
	Order 4	0.393	0.278	0.353	0.145
	Order 5	0.500	0.347	0.435	0.240
Diffuse					
abaxial	Mean	0.640	0.441	0.559	0.253
	Order 1	0.537	0.364	0.463	0.195
	Order 2	0.577	0.386	0.498	0.206
	Order 3	0.943	0.680	0.829	0.405
	Order 4	0.524	0.384	0.482	0.242
	Order 5	0.617	0.391	0.526	0.217
adaxial	Mean	0.755	0.487	0.660	0.250
	Order 1	0.894	0.640	0.820	0.233
	Order 2	0.951	0.680	0.883	0.302
	Order 3	0.449	0.201	0.349	0.182
	Order 4	0.730	0.449	0.621	0.222
	Order 5	0.750	0.466	0.629	0.312
